# The effect of circle of willis anatomy and scanning practices on outcomes for blunt cerebrovascular injuries

**DOI:** 10.1186/s13049-024-01225-x

**Published:** 2024-06-17

**Authors:** David Bar-Or, Stephanie Jarvis, Forrester Lensing, David Bassa, Matthew Carrick, Carlos Palacio Lascano, Maxwell Busch, David Hamilton, David Acuna, Samantha Greenseid, Daniel Ojala

**Affiliations:** 1 Swedish Medical Center, Englewood, CO USA; 2 Saint Anthony Hospital, Colorado, Lakewood, United States; 3 Injury Outcomes Network, Colorado, Englewood, United States; 4 Medical City Plano, Texas, Plano, United States; 5 South Texas Health System McAllen, Texas, McAllen, United States; 6 Penrose Hospital, CO, Colorado Springs, United States; 7 Wesley Medical Center, Kansas, Wichita, United States

**Keywords:** Blunt cerebrovascular injury, Trauma, Scanning practices, Screening criteria

## Abstract

**Background:**

Limited research has explored the effect of Circle of Willis (CoW) anatomy among blunt cerebrovascular injuries (BCVI) on outcomes. It remains unclear if current BCVI screening and scanning practices are sufficient in identification of concomitant COW anomalies and how they affect outcomes.

**Methods:**

This retrospective cohort study included adult traumatic BCVIs at 17 level I-IV trauma centers (08/01/2017-07/31/2021). The objectives were to compare screening criteria, scanning practices, and outcomes among those with and without COW anomalies.

**Results:**

Of 561 BCVIs, 65% were male and the median age was 48 y/o. 17% (*n* = 93) had a CoW anomaly. Compared to those with normal CoW anatomy, those with CoW anomalies had significantly higher rates of any strokes (10% vs. 4%, *p* = 0.04), ICHs (38% vs. 21%, *p* = 0.001), and clinically significant bleed (CSB) before antithrombotic initiation (14% vs. 3%, *p* < 0.0001), respectively. Compared to patients with a normal CoW, those with a CoW anomaly also had ischemic strokes more often after antithrombotic interruption (13% vs. 2%, *p* = 0.02).Patients with CoW anomalies were screened significantly more often because of some other head/neck indication not outlined in BCVI screening criteria than patients with normal CoW anatomy (27% vs. 18%, *p* = 0.04), respectively. Scans identifying CoW anomalies included both the head and neck significantly more often (53% vs. 29%, *p* = 0.0001) than scans identifying normal CoW anatomy, respectively.

**Conclusions:**

While previous studies suggested universal scanning for BCVI detection, this study found patients with BCVI and CoW anomalies had some other head/neck injury not identified as BCVI scanning criteria significantly more than patients with normal CoW which may suggest that BCVI screening across all patients with a head/neck injury may improve the simultaneous detection of CoW and BCVIs. When screening for BCVI, scans including both the head and neck are superior to a single region in detection of concomitant CoW anomalies. Worsened outcomes (strokes, ICH, and clinically significant bleeding before antithrombotic initiation) were observed for patients with CoW anomalies when compared to those with a normal CoW. Those with a CoW anomaly experienced strokes at a higher rate than patients with normal CoW anatomy specifically when antithrombotic therapy was interrupted. This emphasizes the need for stringent antithrombotic therapy regimens among patients with CoW anomalies and may suggest that patients CoW anomalies would benefit from more varying treatment, highlighting the need to include the CoW anatomy when scanning for BCVI.

**Level of Evidence:**

Level III, Prognostic/Epidemiological.

**Supplementary Information:**

The online version contains supplementary material available at 10.1186/s13049-024-01225-x.

## Background

Blunt cerebrovascular injuries (BCVIs) carry a high mortality rate (8-27%); which is the highest for asymptomatic patients, whom often have delayed diagnoses resulting in an increased risk for stroke [1–16]. Concomitant anomalies of the Circle of Willis (CoW) are thought to further increase the stroke risk through reduction of collateral flow [17]. However, in a meta-analysis of six studies including a few different diagnoses such as severe carotid stenosis, ipsilateral internal carotid occlusion, middle cerebral artery occlusion, atherosclerotic diseases, or BCVI, there was a non-significant positive effect of CoW anomalies on the risk of stroke [18]. Two previous studies specifically among patients with BCVI found differing results on the stroke risk, which was dependent on the anatomical region affected or variation type of CoW anomaly [17, 19]. If patients with a CoW anomaly are at an increased risk for stroke, early identification and tailored treatment algorithms may be necessary.

Implementation of BCVI screening criteria has shown to improve BCVI diagnosis rates and outcomes, but studies have not evaluated the correlation between BCVI screening criteria and associated CoW anomalies [2, 4, 6, 11, 13, 14, 20–22]. Universal BCVI screening across all trauma admissions has been suggested to further improve diagnosis rates, but some contend it would be unjustified to scan all trauma patients [12, 16, 23–25]. While the ACS and Scandinavian NeuroTrauma guidelines recommend including the CoW when screening for BCVI, the Eastern Association of Trauma (EAST) and Western Trauma Association (WTA) guidelines not mention the CoW [14, 26–28]. It remains unclear if current BCVI screening criteria are sufficient to capture concomitant CoW anomalies.

When considering the optimal scanning practice to identify BCVI, the CoW is also typically not discussed [3, 6, 12, 14, 20, 24, 26, 27, 29–38]. Major guidelines state that digital subtracted angiography (DSA) is the gold standard for BCVI detection, but recommend the use of computed tomography angiography (CTA) [14, 26, 27]. Improvements to CTA have resulted in comparable BCVI detective rates to DSA, with lower costs and complications, but less accurate grading [3, 12, 14, 20, 26, 27, 29–31, 33, 34, 39]. Magnetic resonance imaging (MRI), magnetic resonance angiography (MRA), and ultrasounds can also be used to identify BCVI, do not use ionizing radiation, account for active arterial flow, but have lower accuracy than CTA, can require sedation, have longer assessment times, and do not have the resolution to capture low-grade BCVIs [6, 31, 35, 40]. Another factor to consider is the region scanned: the head, the neck, or both [24]. In order to capture all CoW anomalies and strokes, both regions should be scanned. The ACS guideline discussed prior studies integrating the neck into a whole-body scan to ensure the CoW is included [40]. The Scandinavian NeuroTrauma Guideline states that a CTA extending through the base of the skull and including the CoW should be included for patients meeting BCVI screening criteria [14]. Because the focus of prior studies has been on BCVI detection, and not CoW identification, this study sought to describe scanning practices identifying CoW anomalies. Prior studies have shown BCVI grades are unlikely to change on repeat imaging, therefore the use and timing of repeat imaging is also debated and varies by guideline [6, 14, 25, 38, 41, 42]. It has been suggested to use repeat imaging to guide treatment, thus it is important to describe changes to BCVI grading over time, and if the vessel involved plays a role.

Little data exists describing the effect of CoW anomalies on outcomes specifically among patients with BCVI. Screening criteria and the best scanning practices (modality, scanner configuration, contrast use, repeat scans, and the body region) associated with CoW identification remain unclear. While prior studies have described how BCVI grades change on repeat imaging summarized by the initial grade, there is a lack of data on BCVI progression summarized by the vessel involved. The study hypotheses were that (1) presence of CoW anomalies would be associated with worsened outcomes, (2) outcomes may vary by the anatomical location of the CoW anomaly (anterior vs. posterior), (3) specific BCVI screening criteria will be associated presence of CoW anomalies, and that meeting any current BCVI scanning indication may not be associated with capturing CoW anomalies, (4) scanning practices would vary for patients with CoW anomalies, and lastly (5) that BCVI progression on repeat imaging would vary by the vessel involved.

## Materials and methods

**Fig. 1:**
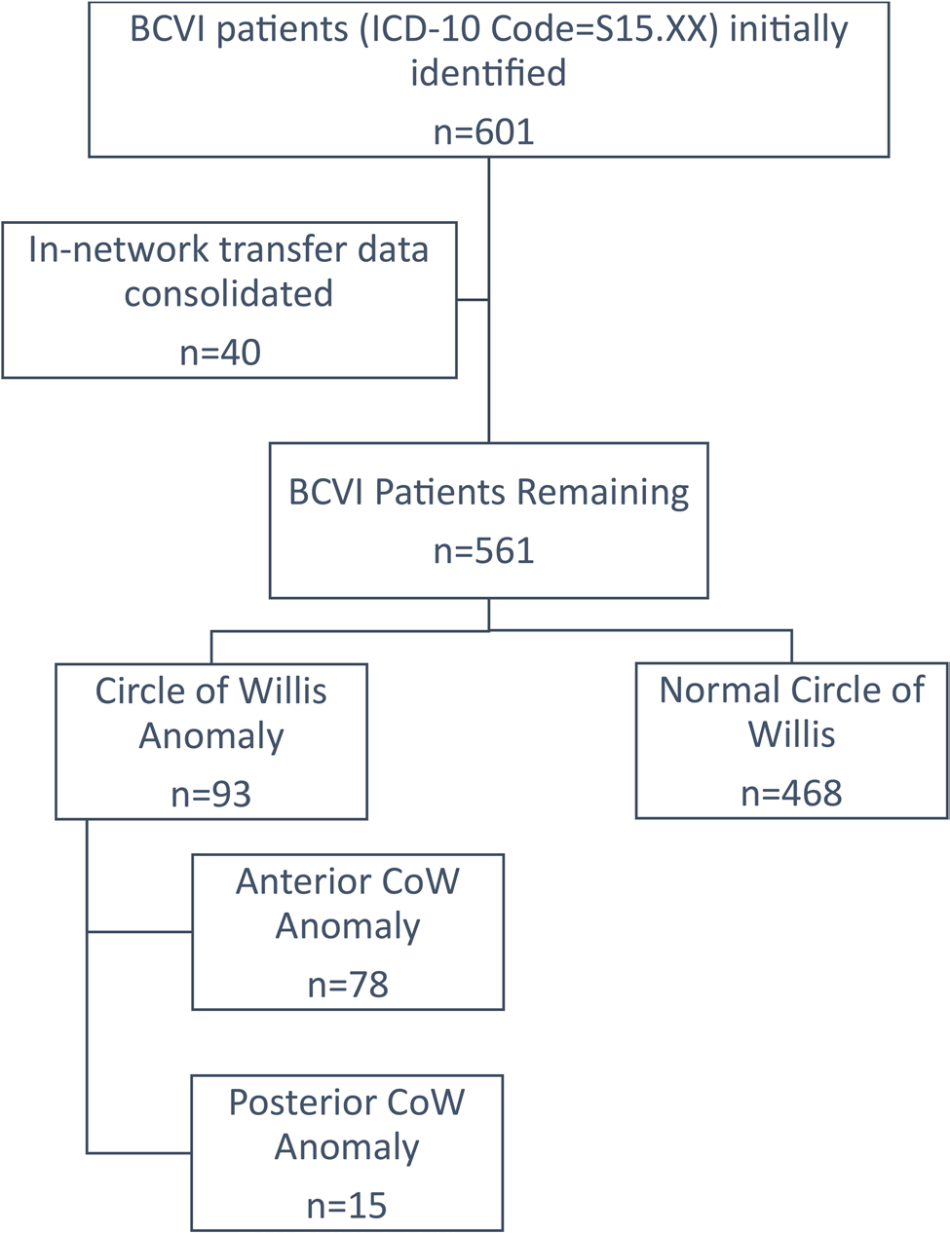
Enrollment flow diagram

This retrospective cohort study within our research consortium of six US Level I and 11 Level II-IV trauma centers included adult trauma patients admitted over four years (8/1/17 − 7/31/21) with an International Classification of Diseases code for BCVI (S15), affecting the carotid (internal, external, common) and vertebral arteries, Fig. 1. In-network transfer data was consolidated so that the patients were not counted twice. First patients with any anomaly to the CoW were compared to those with normal CoW anatomy. Anomalies to the CoW were defined as an anomaly in the intradural internal carotid, posterior cerebral (P1), anterior cerebral (A1), posterior communicating, or anterior communicating arteries. CoW anomalies included those which were missing or absent, unformed, narrowing, incomplete, separations, aneurysms, luminal irregularities, and fetal-type variations. Any CoW anomaly documented in the radiology report was included, imaging was not re-evaluated to identify any missed diagnoses, nor for interrater concordance and discordance. Uniform criteria for identification and diagnosis of CoW anomalies were not followed at the participating centers. Results were further compared by the anatomical location of the CoW variation (anterior vs. posterior). Anterior CoW anomalies included those to the intradural internal carotid, anterior cerebral, and anterior communicating artery. Posterior CoW anomalies included those affecting the posterior cerebral arteries or posterior communicating artery. This study was approved by the participating center’s institutional review board with a waiver of patient consent.

Figure 1 is a flow diagram describing the patient enrollment and comparison groups for this study. Data were collected from the participating center’s trauma registry and electronic health records (EHR). EHR data were entered into Research Electronic Data Capture (REDCap®). Baseline demographics and clinical characteristics were collected to describe any differences in characteristics among patients with and without COW anomalies and describe any potential confounding, effect modification, or interaction. These variables included sex, age, race, ethnicity, cause of injury, National Trauma Data Standard (NTDS) comorbidities, injury severity scale (ISS), as well as preinjury anticoagulant or antiplatelet use. Additional variables assessed but not presented in this manuscript included the following: weight, height, body mass index, admission vitals [temperature, heart rate, respiratory rate, systolic blood pressure, diastolic blood pressure, oxygen saturation, and Glasgow Coma Scale (GCS)], trauma activation level, and maximum abbreviated injury scale by location; these variables did not vary when compared by presence of CoW anomalies. Scanning methods collected included: modality [CTA, MRA, MRI, DSA, four vessel cerebral angiography (FVCA), ultrasound], contrast use, scanner configuration, and region scanned [head, neck, head and neck, as part of a whole-body scan). The scanner configuration and utilization at the participating centers is summarized in Supplemental Fig. 1. The primary outcome was stroke (ischemic, hemorrhagic, or either). Other clinical outcomes included: ICH, clinically significant bleeding (CSB), BCVI grade progression (improved, worsened, no change), hospital length of stay (HLOS), intensive care unit length of stay (ICULOS), in-hospital mortality, and discharge disposition (home or home with health services, hospice, long-term acute care, rehabilitation, skilled nursing facility, other). Any CSB included gastrointestinal bleeding, expanding hematomas, bleeding at the site of blunt solid organ injury, retroperitoneal hemorrhage, surgical, or bleeding that resulted in actionable treatment (i.e., blood transfusion, surgery). Any bleeding complication (ICH or CSB) were analyzed together and separately for sensitivity analyses. Further sensitivity analyses were conducted by summarizing the CSB by the timing in relation to antithrombotic administration (*before* antithrombotic initiation, *after*, or *both*) and by summarizing the rate of IS within the various populations (patients with ICH, CSB, antithrombotic medications administered). BCVI grading, using the Denver grading system, was summarized by the artery involved (carotid, vertebral), and side affected (left, right). In brief, Grade I injuries include irregularity of the vessel wall or dissection/intramural hematoma with < 25% luminal stenosis, Grade II injuries include intraluminal thrombus or raised intimal flap or dissection/intramural hematoma with > 25% luminal stenosis, Grade III are pseudoaneurysms, Grade IV are occlusions, and Grade V are transections [34]. The rate of antithrombotic administration (which included aspirin, clopidogrel, enoxaparin, heparin, warfarin, apixaban, and rivaroxaban) and the rate of antithrombotic interruption was described. Data missing from the registry were abstracted from the electronic medical record. Any variable that was missing from the registry and was not in the electronic medical record was labelled as unknown or undetermined in the tables.

Supplemental Fig. 1 summarizes the number of trauma centers with each scanner configuration and whether that trauma center used that CT-slice for the patients enrolled in this study. The black bars indicate that the scanner configuration was available and used, the dark grey indicates that the scanner configuration was available but not used for the patients included in this study, and the light grey indicates the scanner configuration was not available.

The indication to scan is documented in the radiology reports at the participating centers. BCVI scanning criteria were abstracted from the patient’s radiology reports. If other reasons for scanning not included as a BCVI scanning criteria were dictated, the information on why the scan was conducted was collected. Other reasons for scanning were consolidated during analysis into the following categories: trauma admission scanning, suspected BCVI, BCVI follow-up, or some other head/neck injury not currently included in guidelines as BCVI scanning criteria.

Our group previously published a separate study aimed at comparing the effects of antithrombotic regimens (type, dose, frequency) on outcomes among patients with BCVI [43]. The detailed antithrombotic practices at the participating centers for BCVI were described in detail [43]. The purpose of this study was not to examine the effects of antithrombotic therapy on outcomes, because of this the collection of antithrombotic data was simplified; only the use of antithrombotic medications and if antithrombotic therapy was interrupted were collected. Interruption of antithrombotic therapy was defined as an unplanned missed dose. Interrupted therapy was identified in progress notes, through physicians’ orders to hold antithrombotic medication, or by a clear missed dose in the patient’s medication history.

Statistical Analysis System 9.4 (SAS Institute, Cary NC) was used for statistical analysis. Data were summarized as means with standard deviation (SD), median with interquartile range (IQR), or proportion (confidence interval). Confidence intervals were calculated using the Wilson method which is appropriate and stable for small sample sizes. Fisher’s exact test, chi-squared test, Student’s t-test, and Wilcoxon rank sum test were used. An alpha of less than 0.05 defined statistically significant differences. The equator STROBE checklist was followed for reporting standards. The aims and results described in this manuscript are secondary aims of a larger descriptive study focusing on scanning practices for BCVI. The sample size estimate for the primary aim was 600 patients, which was based on a convenience sample estimate using the number of BCVI patients admitted to each hospital annually. A sample size was not calculated for these secondary aims. Because of the small sample size of CoW anomalies and rarity of strokes, adjusted regression analyses could not be conducted. Instead, the variables identified as potential confounders were described in detail. The variables which were significantly different when compared by presence of CoW anomalies were then compared by presence of stroke to identify potential confounders, effect modifiers or interactions, which were then described in detail.

## Results

Of the 561 traumatic BCVIs, a majority were male (65%), predominantly White (77%), with a median age of 48 years/old, Table 1. There were 93 patients with CoW anomalies, 13% (71) had an abnormal intradural internal carotid artery, 2% (12) had an abnormal posterior cerebral artery, 1% (5) had an abnormal anterior cerebral artery, 1% (5) had an abnormal anterior communicating artery, and 1% (3) had an abnormal posterior communicating artery. Patients with CoW anomalies were significantly younger (41 vs. 49, *p* = 0.002) than patients with normal CoW anatomy. Those with CoW anomalies suffered falls significantly less often (17% vs. 28%, *p* = 0.01) than patients with normal CoW anatomy, respectively. Patients with CoW anomalies had diabetes significantly less often (2% vs. 9%, *p* = 0.03) and prior myocardial infarctions (MI) significantly more often (2% vs. 0%, *p* = 0.03) than patients with normal CoW anatomy, respectively, other comorbidities were similar between groups. The ISS was significantly higher for those with CoW anomalies than those with normal CoW anatomy, (21 vs. 17, *p* = 0.003), respectively.

**Table 1:** Patient demographics and characteristics

	All Patients *n* = 561	Circle of Willis Anomaly *n* = 93	Circle of Willis Normal *n* = 468	*p*
**Sex**, % Male (n)	64.7% (363)	65.6% (55.5, 74.5)	64.5% (60.1, 68.7)	0.91
**Age**, Median (IQR)*	48.0 (32.0, 64.0)	41.0 (28.0, 57.0)	49.0 (33.0, 66.0)	**0.002**
**Age ≥ 89 years old**, % (n)	2.1% (12)	0% (0, 3.2)	2.6% (1.5–4.4)	0.23
**Race**, % (n)				
American Indian	0.7% (4)	0% (0-3.2)	0.9% (0.3–2.2)	
Asian	1.3% (7)	2.2% (0.6–7.8)	1.1% (0.5–2.5)	
Black or African American	4.3% (24)	5.4% (2.3–12.0)	4.1% (2.6–6.3)	0.73
Other/Unknown	17.3% (97)	16.1% (10.0-24.9)	17.5% (14.4–21.2)	
White	76.5% (429)	76.3% (66.8–83.8)	76.5% (72.5–80.1)	
Hispanic, % (n)	8.2% (46)	10.8% (6.0-18.7)	7.7% (5.6–10.5)	0.55
**Cause of Injury**, % (n)				
MCC/MVC	48.8% (274)	48.4% (38.5–58.4)	48.9% (44.4–53.5)	
Fall	26.6% (149)	17.2% (10.9–26.1)	28.4% (24.5–32.7)	**0.01**
Other/Unknown	21.4% (120)	25.8% (18.0-35.5)	20.5% (17.1–24.4)	
Assault	1.8% (10)	4.3% (0.6–2.8)	1.3% (1.7–10.5)	
Gunshot Wound	1.4% (8)	4.3% (1.7–10.5)	0.9% (0.3–2.2)	
**Comorbidities**, % (n)				
Smoker	21.9% (123)	22.6% (15.3–32.1)	21.8% (18.3–25.8)	0.87
Hypertension	21.3% (120)	14.0% (8.4–22.5)	22.9% (19.3–26.9)	0.07
Alcoholism	9.1% (51)	14.0% (8.4–22.5)	8.1% (6.0–11.0)	0.07
Diabetes	7.5% (42)	2.2% (0.6–7.6)	8.6% (6.3–11.4)	**0.03**
Peripheral Artery Disease	0.7% (4)	1.1% (0.2–5.8)	0.6% (0.2–1.9)	0.52
Myocardial Infarction	0.4% (2)	2.2% (0.6–7.6)	0% (0-0.6)	**0.03**
Injury Severity Scale, Median (IQR)	17.0 (10.0, 26.0)	21.0 (13.0, 29.0)	17.0 (9.0, 25.0)	**0.003**
Pre-injury Anticoagulants, % (CI)	4.1% (23)	1.1% (0.2–5.8)	4.7% (3.1-7.0)	0.15
Pre-injury Antiplatelets, % (CI)	8.7% (49)	4.3% (1.7–10.5)	9.6% (7.3–12.6)	0.31

*Where age < 89 years old, age older than 89 is coded due to data use restrictions with participating hospitals. BMI: body mass index. CI: confidence interval. IQR: interquartile range. MCC/MVC: motorcycle collision/motor vehicle collision, COPD: chronic obstructive pulmonary disease. Circle of Willis (COW) anomalies were compared to normal COW anatomy and included those which were missing or absent, unformed, narrowing, incomplete, separations, aneurysms, luminal irregularities, and fetal-type variations. Bold p-values indicate statistical significance

### Outcomes

Patients who had CoW anomalies suffered any stroke (both hemorrhagic and ischemic) significantly more often than patients with a normal CoW (10% vs. 4%, *p* = 0.04), despite having similar rates of antithrombotic administration (91% vs. 90%, *p* = 0.70), respectively, Table 2. Those with CoW anomalies also suffered an ICH (38% vs. 21%, *p* = 0.001) or other CSBs (29% vs. 14%, *p* = 0.001) significantly more often than patients with normal CoW anatomy, respectively. There was no difference in the rate of ischemic stroke when compared by presence of CoW anomaly among those with antithrombotic medications administered (*p* = 0.25), however there was a significantly higher rate of ICH among patients with a CoW anomaly when compared to normal CoW anatomy (38% vs. 19%, *p* = 0.0003). Among patients who did not receive in-hospital antithrombotic therapy, those who had a CoW anomaly had a higher rate of ischemic stroke than those with a normal CoW, but the difference was not significant (13% vs. 2%, *p* = 0.26). Furthermore, among patients whose antithrombotic therapy was interrupted, there was also a higher rate of ischemic strokes for patients with a CoW anomaly than for those with a normal CoW (13% vs. 2%, *p* = 0.02). Those with CoW anomalies experienced CSB *before* antithrombotic administration significantly more often (14% vs. 3%, *p* < 0.001), than patients with normal CoW anatomy, respectively. Among patients with CSB *after* antithrombotic administration, there was also a significantly higher rate of ischemic stroke for patients with CoW anomalies when compared to those with a normal COW (57% vs. 17%, *p* = 0.045), respectively. The stroke rate (*p* = 0.34) was similar when examined by anatomical location of CoW variations, anterior vs. posterior. Patients with anterior anomalies had CSB *after* antithrombotic initiation significantly less often than those with a posterior anomaly (4% vs. 23%, *p* = 0.04), respectively. While the ICH rate was higher for those with anterior anomalies than posterior anomalies, it was not statistically significant (*p* = 0.12). ICULOS (5 vs. 2, *p* = 0.01) was significantly longer for those with an anterior anomaly than for those with a posterior anomaly, respectively.

**Table 2:** Circle of willis anatomy and outcomes

	All BCVIs *n* = 561 % (*n*/*N*)	Circle of Willis Anomaly 16.6% (93/561) % (95% CI)	Circle of Willis Normal 83.4% (468/561) % (95% CI)	*p*	Anterior Anomaly 83.9% (78/93) % (95% CI)	Posterior Anomaly 16.1% (15/93) % (95% CI)	*p*	
**Any Stroke**	5.4% (29/561)	9.7% (5.2–17.4)	4.3% (2.8–6.5)	**0.04**	9.0% (4.4–17.4)	13.3% (3.7–37.9)	0.63	
Hemorrhagic Stroke	0.7% (4/561)	2.2% (0.6–7.5)	0.4% (0.1–1.5)	0.13	1.3% (0.2–6.9)	6.7% (1.2–29.8)	0.19	
Ischemic Stroke (IS)	4.5% (25/561)	7.5% (3.7–14.7)	3.9% (2.5-6.0)	0.16	7.7% (3.6–15.8)	6.7% (1.2–29.8)	> 0.99	
**Any Bleeding Complication**	47.3% (44/561)	47.3% (37.5–57.4)	29.5% (25.5–33.8)	**0.001**	47.4% (36.7–58.4)	41.7% (19.3–68.1)	> 0.99	
ICH	24.1% (135/561)	37.6% (28.5–47.8)	21.4% (17.9–25.3)	**0.001**	41.0% (30.8–52.1)	20.0% (7.1–45.2)	0.12	
IS in Patients with an ICH	2.2% (2/135)	5.7% (1.6–18.6)	1.0% (0.2–5.5)	0.16	6.3% (1.7, 20.2)	0% (0-16.7)	> 0.99	
Other Clinically Significant Bleed (CSB)	16.0% (72/451)	29.0% (19.6–40.6)	13.6% (10.5–17.4)	**0.001**	26.8% (17.0-39.6)	38.5% (17.7–64.4)	0.50	
IS in Patients with Other CSB	19.4% (14/72)	25.0% (11.2–48.9)	17.3% (9.4–29.7)	0.51	26.7% (10.9–52.0)	20.0% (3.6–62.5)	> 0.99	
**Antithrombotic Administered**	89.8% (503)	91.4% (83.0-95.6)	89.5% (86.4–92.0)	0.70	92.3% (84.2–96.4)	86.7% (62.1–96.3)	0.61	
IS in those Given Antithrombotics	4.6% (23/503)	7.1% (3.3–14.6)	4.1% (2.6–6.4)	0.25	6.9% (3.0-15.3)	7.7% (1.4–33.3)	> 0.99	
IS in those Not Given Antithrombotics	3.5% (2/57)	12.5% (2.2–47.1)	2.0% (0.4–10.7)	0.26	16.7% (3.0-56.4)	0% (0–25.0)	> 0.99	
ICH in those Given Antithrombotics	21.9% (110/503)	37.7% (28.1–48.3)	18.7% (15.2–22.7)	**0.0003**	41.0% (30.8–52.1)	15.4% (4.3–42.2)	0.12	
ICH in those Not Given Antithrombotics	42.1% (24/57)	37.5% (13.7–69.4)	42.9% (30.0-56.7)	> 0.99	50.0% (9.5–90.6)	33.3% (9.7–70.0)	> 0.99	
**Antithrombotics Interrupted**	31.2% (157/503)	26.5% (18.2–36.9)	32.2% (27.9–36.9)	0.40	24.7% (15.8–35.5)	38.5% (17.7–64.4)	0.32	
IS in those with Antithrombotics Interrupted	3.2% (5/157)	13.0% (4.5–32.1)	1.5% (0.4–5.3)	**0.02**	11.1% (3.1–32.8)	20.0% (3.6–62.5)	0.54	
IS in those without Antithrombotic Interrupted	5.2% (16/368)	4.6% (1.6, 1.7)	5.3% (3.3–8.4)	> 0.99	5.5% (1.9 14.9)	0% (0–5.0)	> 0.99	
CSB Before Antithrombotics Administered	5.6% (28/503)	14.0% (9.2–24.4)	3.2% (2.2–5.8)	**< 0.0001**	15.3% (8.8–25.3)	15.4% (4.3–42.2)	> 0.99	
IS in those with Bleed Before Antithrombotics	21.4% (6/28)	7.7% (1.4–33.3)	33.3% (15.2–58.3)	0.17	9.1% (1.6–37.7)	0% (0–25.0)	> 0.99	
CSB After Antithrombotics Administered	7.2% (37/503)	7.1% (3.3–14.6)	7.2% (5.1–10.1)	0.70	4.2% (1.4–11.6)	23.1% (8.2–50.3)	**0.04**	
IS in those with Bleed After Antithrombotics	24.3% (9/37)	57.1% (25.1–84.2)	16.7% (7.3–33.6)	**0.045**	75.0% (30.1–95.4)	33.3% (6.2–79.2)	0.49	
CSB Both Before and After Administered	1.4% (8/503)	0% (0-3.2)	1.7% (0.9–3.3)	0.36	0% (0-0.6)	0% (0-3.8)	N/A	
IS in those with Bleed Before and After	0% (0/8)	0% (0-3.2)	0% (0-0.6)	N/A	0% (0-0.6)	0% (0-3.8)	N/A	
HLOS, Median Days (IQR)	7.0 (3.0, 13.1)	7.0 (3.0, 14.0)	7.0 (3.0, 13.0)	0.66	8.0 (4.0, 14.0)	5.0 (3.0, 8.0)	0.052	
ICULOS, Median Days (IQR)	3.0 (1.0, 7.0)	4.0 (2.0, 8.0)	3.0 (1.0, 7.0)	0.16	5.0 (2.0, 10.0)	2.0 (0, 3.0)	**0.03**	
Mortality	7.3% (41/561)	8.6% (4.4–16.1)	7.1% (5.1–9.7)	0.66	7.7% (3.6–15.8)	13.3% (3.7–37.9)	0.61	

ICH: intracerebral hemorrhage, CSB: clinically significant bleed, HLOS: hospital length of stay, ICULOS: intensive care unit length of stay. *Unless otherwise specified in the parentheses next to the percentage, the denominator for the first column is 561. The anterior artery includes the internal carotid artery, anterior cerebral artery, and anterior communicating artery. The posterior includes the posterior cerebral artery and the posterior community artery. The circle of Willis anomalies included those which were missing or absent, unformed, narrowing, incomplete, separations, aneurysms, luminal irregularities, and fetal-type variations. Bleeding complications included clinically significant bleeding requiring any actionable treatment. Bold p-values indicate statistical significant differences between groups

### Screening criteria

The index scan identifying a BCVI was often (31%) conducted for some other reason *not* outlined as BCVI screening criteria, including (1) trauma admission scanning (22%) or having some (2) other head/neck injury (12%), Table 3. Patients with CoW anomalies met BCVI scanning criteria prompting the scan significantly less often than patients with a normal CoW (54% vs. 72%, respectively, *p* = 0.0004). Patients with CoW anomalies were scanned for some other head/neck injury not outlined as BCVI scanning criteria significantly more often than patients with normal COW anatomy (18% vs. 11%, *p* = 0.04), respectively. However, there were some BCVI screening criteria which were more common among patients with CoW anomalies than those with a normal CoW including complex skull fractures (13% vs. 6%, *p* = 0.03), severe traumatic brain injuries with a GCS < 6 (16% vs. 4%, *p* < 0.0001), or mandible fractures (7% vs. 2%, *p* = 0.03), respectively.

**Table 3:** Scanning criteria or other reasons for scanning

	BCVI Index Scan 100% (561) % (*n*)	COW Anomaly 16.6% (93) % (95% CI)	COW Normal 83.4% (468) % (95% CI)	*p*
Met Any Guideline Screening Criteria	69.3% (389)	53.8% (44.7–64.6)	72.4% (70.0-77.9)	**0.0004**
Cervical spine fracture^a^	42.3% (237)	15.2% (9.3–23.9)	47.7% (43.2–52.2)	**< 0.0001**
TBI with thoracic injury	11.1% (62)	2.2% (0.6–7.6)	12.8% (10.1–16.2)	**0.002**
Upper rib fracture	8.9% (50)	8.6% (4.5–16.2)	9.0% (6.7–11.9)	0.91
Complex skull fracture^b^	7.5% (42)	12.9% (8.5–22.7)	6.4% (4.5-9.0)	**0.03**
Severe TBI with GCS < 6	6.1% (34)	16.1% (10.1–25.2)	4.1% (2.6–6.3)	**< 0.0001**
Focal neurologic defect^c^	4.1% (23)	5.4% (3.0-13.5)	3.9% (2.5-6.0)	0.56
Clothesline type injury^d^	4.1% (23)	2.2% (0.6–7.6)	4.5% (3.0-6.8)	0.40
Neurologic deficit inconsistent with head CT	3.2% (18)	1.1% (0.2–5.9)	3.6% (2.3–5.7)	0.33
Mandible fracture	2.9% (16)	6.5% (3.0-13.5)	2.1% (1.2–3.9)	**0.03**
Thoracic vascular injuries	2.0% (11)	1.1% (0.2–5.9)	2.1% (1.2–3.9)	0.70
Near hanging with anoxic brain injury	1.4% (8)	1.1% (0.2–5.9)	1.5% (0.7–3.1)	> 0.99
Displaced midface fracture (LeFort II or III)	1.3% (7)	2.2% (0.6–7.6)	1.1% (0.5–2.5)	0.33
Stroke on CT or MRI	0.9% (5)	1.1% (0.2–5.9)	0.9% (0.3–2.2)	> 0.99
Cervical bruit in patient < 50 years old	0.5% (3)	1.1% (0.2–5.9)	0.4% (0.1–1.5)	0.42
Expanding cervical hematoma	0.5% (3)	0% (0-3.2)	0.6% (0.2–1.9)	> 0.999
Arterial hemorrhage from neck/nose/mouth	0.2% (1)	0% (0-3.2)	0.2% (0.04–1.2)	> 0.99
Scalp degloving	0.2% (1)	0% (0-3.2)	0.2% (0.04–1.2)	> 0.99
Blunt cardiac rupture	0% (0)	0% (0-3.2)	0% (0-6.4)	N/A
**Other Reason for Scanning**	30.7% (172)	46.2% (36.5–56.3)	27.6% (23.7–31.8)	**0.0004**
Trauma admission scanning	22.3% (125)	21.5% (14.4–30.9)	22.4% (18.9–26.4)	0.84
BCVI Suspected	0.2% (1)	0% (0-3.2)	0.2% (0.04–1.2)	> 0.99
BCVI F/U	0.2% (1)^e^	0% (0-3.2)	0.2% (0.04–1.2)	> 0.99
Other Head or Neck Injury	12.1% (68)	18.3% (11.7–27.3)	10.9% (8.4–14.1)	**0.04**

CI: confidence interval, a = or subluxation, or ligamentous injury at any level. b = or basilar skull fracture or occipital condyle fracture. c = hemiparesis, vertebrobasilar symptoms, Horner syndrome, transient ischemic attack. d = or seat belt abrasion with significant swelling, pain, or altered mental status. e = One patient transferred in to one of the enrolling facilities received their index scan as a follow-up scan, original scan information from the index facility was not available. COW: circle of Willis, CT: computed tomography, MRI: magnetic resonance imaging, TBI: traumatic brain injury, GCS: Glasgow coma scale, BCVI: blunt cerebrovascular injury, F/U: follow up. The screening criteria used for patients with COW anomalies for the index BCVI scan was compared to the screening criteria indicating to scan for patients with normal COW anatomy. COW anomalies included those which were missing or absent, unformed, narrowing, incomplete, separations, aneurysms, luminal irregularities, and fetal-type variations. Bold p-values indicate statistical significant differences between groups

### Scanning methods

The index scan identifying a BCVI was almost always a 64-slice CTA during a whole-body scan, that included the neck, Table 4. Compared to scans identifying a normal CoW, scans identifying BCVIs with concomitant CoW anomalies were of the head and neck only significantly more often (53% vs. 29%, *p* = 0.0001), were part of a whole-body scan significantly less often (25% vs. 42%, *p* = 0.002) and used a 64-slice CT significantly more often (82% vs. 72%, *p* = 0.002).

**Table 4:** Scanning practices identifying BCVI, COW anomalies, and strokes

	Index BCVI Diagnosis n = 561 % (n)	Scan Identifying COW Anomalies BCVI Diagnosis n = 93 % (95 % CI)	Index Scan with COW Normal n = 468 % (95% CI)	p
**Scan Type**				
Computed Tomography Angiography	95.7% (537)	96.8% (94.0-99.8)	96.5% (96.3–99.0)	**0.38**
Computed Tomography	2.5% (14)	1.1% (0.6–7.5)	2.6% (1.5–4.4)	
Magnetic Resonance Imaging	1.6% (9)	1.1% (0.2–5.8)	1.7% (0.9–3.3)	
Magnetic Resonance Angiography	0.2% (1)	0% (0-3.2)	0.2% (0.2–43.5)	
Four Vessel Cerebral Angiography	0% (0)	1.1% (0.6–7.8)	0% (0-0.6)	
Digital Subtraction Angiography	0% (0)	0% (0-3.2)	0% (0-0.6)	
Ultrasound	0% (0)	0% (0-3.2)	0% (0-0.6)	
**Contrast Used**	97.7% (548)	97.9% (94.2–99.8)	97.4% (95.6–98.5)	> 0.99
**CT-Slice, for CTs/CTAs** ^1^				
16	3.6% (20)	4.6% (1.8–11.1)	3.6% (2.2–5.7)	**0.002**
32	1.3% (7)	4.6% (1.8–11.1)	0.7% (0.2–1.9)	
64	71.6% (395)	81.6% (72.5–88.5)	71.8% (67.5–75.7)	
128	20.5% (113)	9.2% (4.7–16.9)	23.3% (19.7–27.5)	
512	0.5% (3)	0% (0-3.2)	0.7% (0.2–1.9)	
**Region Scanned**				
Head and Neck only	20.4% (115)	52.9% (39.2–62.2)	29.3% (24.2–35.0)	**0.001**
Head only	1.1% (6)	0% (0-3.2)	2.2% (1.0-4.7)	
Neck only	39.4% (221)	47.1% (37.8–60.8)	68.5% (62.8–73.7)	
**During Whole-Body Scan**	38.8% (218)	24.7% (18.0-35.5)	41.5% (37.2–46.1)	**0.002**
Head and Neck Included^2^	12.8% (72)	39.1% (21.2–57.3)	32.5% (26.3–39.4)	**0.01**
Head included^2^	0.4% (2)	8.7% (2.3–25.9)	0% (0-0.6)	
Neck included^2^	26.0% (144)	52.2% (35.1–72.1)	67.5% (60.7–73.7)	

BCVI: Blunt cerebrovascular injury, CI: confidence interval, CT: computed tomography, CTA: computed tomography angiography. 1 = There were 10 scans for transfer patients with an unknown slice-count on the first BCVI scan, all of these scans occurred at the index facility before transferring to one of the participating centers. 2=During whole-body scan. Bold p-values indicate statistical significant differences between groups

### BCVI grading and progression

**Fig. 2:**
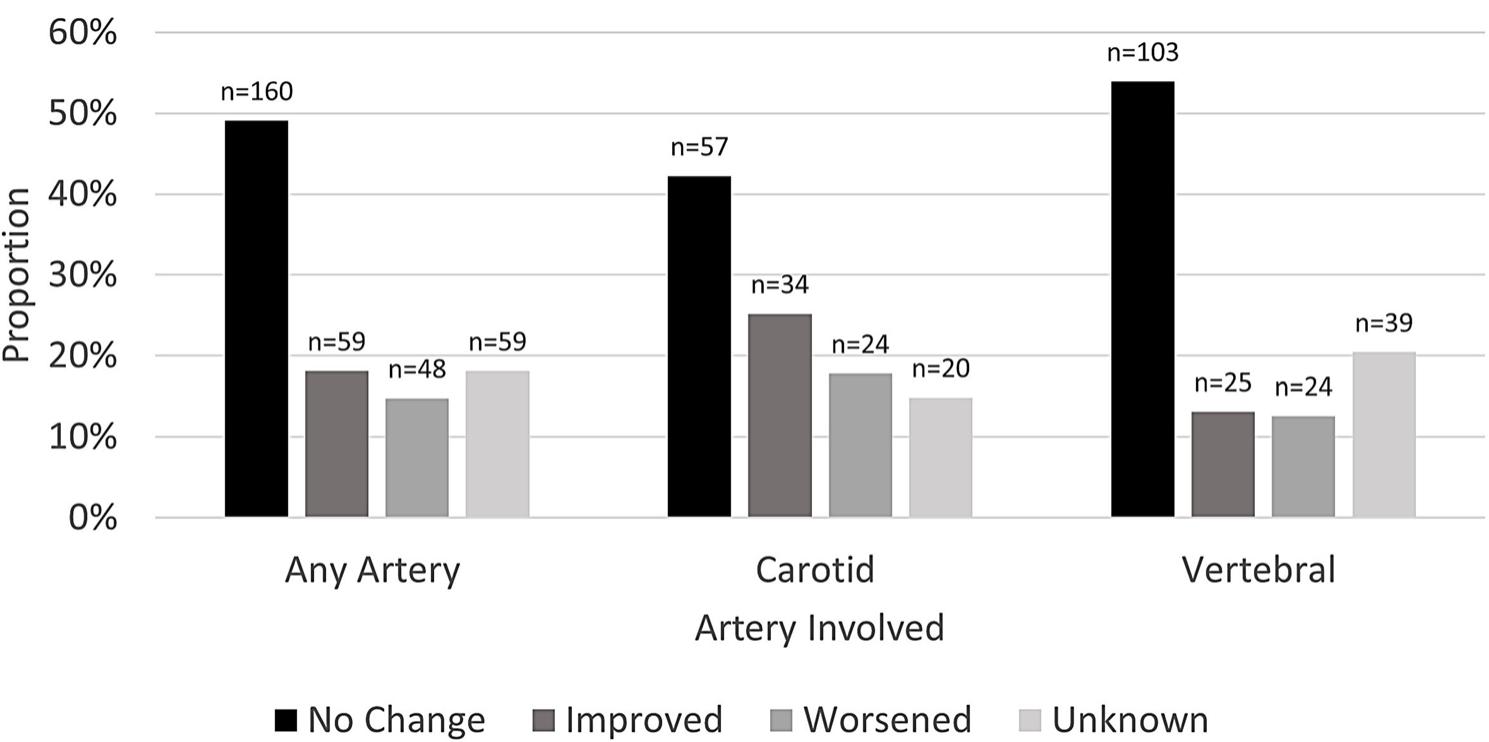
BCVI Progression for patients with repeat imaging

BCVI grading described by the vessel involved initially, and on every subsequent scan identifying a BCVI can be seen in Supplemental Table 1. In general, left or right vertebral injuries (41–44%) occurred more than left or right carotid injuries (25–26%). When looking at the highest grade determined during hospitalization, carotid injuries often had lower grade injuries (I-II) than vertebral injuries, representing irregularities in the vessel walls, intramural hematomas with luminal stenosis, intraluminal thrombus, or a raised intimal flap. Vertebral injuries had higher rates of grade IV injuries (occlusions) than carotid injuries. The progression of BCVI grades on repeat imaging is described in Fig. 2, for the 56% of patients with multiple scans identifying a BCVI. Only 17% experienced a worsening grade. Carotid injuries had higher rates of improved grades (27% vs. 15%) and had higher rates of worsening injuries (20% vs. 14%) than vertebral injuries, respectively. Patients with vertebral artery injuries had higher rates of no change (60% vs. 50%) in their grade or unknown progressions (25% vs.16%) than carotid artery injuries, respectively. Subsequent scans diagnosing newly detected BCVIs not seen on the index scan identifying a BCVI were infrequent (11% of those with repeat imaging, *n* = 22/208), but often patients had multiple newly detected BCVIs (40%, *n* = 9) (data not tabulated). Four patients with CoW anomalies had six newly detected BCVIs, there were no significant differences in the rate of newly detected BCVIs among those with and without CoW anomalies (15% vs. 10%, *p* = 0.44, respectively). The frequency of each vessel involved for patients with newly detected BCVIs was as follows: left vertebral, 41% (*n* = 9/22), right carotid, 36% (*n* = 8/22), right vertebral, 32% (*n* = 7/22), and left carotid artery, 32% (*n* = 7/22).

Figure 2 displays the BCVI progression for patients who had at least two scans identifying a BCVI (*n* = 208/561; 37%), representing a total of 326 BCVIs. The progression is displayed for all artery injuries and stratified by the artery involved. Only scans with BCVIs reported were included in this study, thus BCVIs which were completely resolved were not captured and are not included in the improved group. It is presented as the proportion of arteries with no change (black) in their BCVI grade, the proportion of arteries with an improved (darkest grey) BCVI grade on any scan during hospitalization, the proportion of arteries with a worsening (middle grey) BCVI grade during hospitalization, or the proportion of arteries who had an unknown progression (lightest grey) because the index or subsequent grade was unable to be determined.

## Discussion

This study observed worsened outcomes among patients with CoW anomalies, which highlights the need to include the CoW in BCVI screening and diagnostic studies. This may suggest that varying treatment is warranted for BCVI patients with concomitant CoW anomalies, but future studies are needed to confirm this. While both screening and scanning criteria for BCVI diagnoses have been well researched, there is a lack of information on screening and scanning practices that are useful for identification of CoW anomalies. There were patterns identified for both screening and scanning practices useful in identification of CoW in this study across 17 trauma centers which were not following uniform practices. Three specific BCVI screening criteria were associated with patients with CoW anomalies, and importantly, patients with CoW anomalies had a scan conducted for some other reason not currently identified as a BCVI screening criteria more often than patients with a normal CoW. Thus, current BCVI screening criteria are not inclusive of identifying co-occurring CoW anomalies. CoW anomalies were also identified more often when both the head and neck were included during the scan, rather than only one region or during a whole-body scan.

Having a complete CoW is thought to be protective of stroke as the CoW can respond to low perfusion pressure by reversing the flow providing collateralization after BCVI [17, 19, 44]. In this study a specific subset of BCVI patients with CoW anomalies were not only significantly more likely to suffer strokes, but were also more likely to have ICHs, and CSB before antithrombotic initiation than those with normal CoW anatomy. This could be a result of the altered collateral flow. This emphasizes the need to include the CoW in BCVI imaging studies and potentially the need for tailored treatment. Currently antithrombotic therapy is the frontline therapy to prevent strokes, but antithrombotic use must be balanced to consider the risk of CSB and ICH [22, 43, 45, 46]. Future studies are needed to help determine the optimal treatment for these patients, or identification of patients who would benefit from what treatment: antithrombotic or endovascular therapy. A prior study observed that the time from arrival to antithrombotic administration was significantly longer for patients who later developed an ICH, and that antithrombotic interruption was associated with a higher stroke rate [43]. Those two treatment factors may be even more important for patients with CoW anomalies. While the use of surgery is rare for BCVI, stent placement has become more common, however current indications for stenting are unclear [47]. Brommeland et al. state high grade injuries or pseudoaneurysms may be considered for endovascular therapy; evaluation of the use of endovascular therapies (stent placement, coil embolization) in patients with CoW anomalies may be warranted [14, 47]. In contrast to our findings, another study with a small sample size observed patients with normal CoW anatomy were 5.8 times more likely to have a stroke than patients with CoW anomalies [17]. Lee et al. found CoW variations in the internal carotid artery were associated with an increased risk of stroke during endovascular clamping [19]. Zhou et al. conducted a study of patients with cerebrovascular diseases and observed that having a CoW anomaly was associated with worsened National Institutes of Health Stroke Severity (NIHSS) scores when compared to having a normal CoW [48]. Another study found no difference in the rate of early neurologic events, including stroke, after carotid eversion endarterectomy when compared by presence of CoW anomalies [49]. Murphy et al. stressed the need for more studies evaluating the risks and benefits of treatment among BCVI patients, stating that delayed treatment, or no treatment may result in stroke, whereas antithrombotic therapy may result in bleeding or head injury progression among patients with BCVI [50]. Balancing the risks and benefits of antithrombotic therapy can be difficult for physicians when there is little data available, but this study was successful in identifying a specific subset patients with BCVI who may benefit from more tailored or aggressive treatment, being those with CoW anomalies, however further studies are still needed to confirm this.

Despite the fact that those with and without CoW anomalies received similar rates of antithrombotic administration during hospitalization, patients with a CoW anomaly still had higher rates of strokes, suggesting more aggressive treatment may be warranted for those with both BCVI and CoW anomalies. Furthermore, among patients *with* antithrombotic therapy interrupted, the stroke rate was significantly higher among patients with a CoW anomaly, whereas among those *without* antithrombotic interruption there was no difference in the stroke rate when compared by presence of CoW anomalies. This highlights the importance of antithrombotic therapy and may suggests that tailored treatment may be necessary to prevent strokes, which may include entail earlier antithrombotic initiation, more aggressive antithrombotic regimens, or even endovascular therapy. While the EAST guideline recommends the use of antithrombotic therapy, they state there currently is not enough data to support recommendations on the type, timing of initiation, dose, or duration of therapy [27]. WTA provides guidance that those with Grade I-IV injuries be provided low molecular weight heparin with a goal of a partial thromboplastin time of 40–50 s, stating that low molecular weight heparin is preferred as it is reversible and may be more efficacious than other antiplatelet drugs [28]. Further the WTA recommends endovascular treatment for Grade V BCVIs [28]. The Scandinavian Journal of Trauma recommends early antithrombotic treatment with heparin (50–100 IU/kg twice daily) within the first 24–48 h of diagnosis, then to transfer to oral acetyl salicylic acid (75 mg daily) and to continue treatment for at least three months [14]. They state that patients with severe luminal stenosis or progressing pseudoaneurysms should be consulted for potential endovascular therapy [14]. Future studies evaluating the efficacy of various treatment methods for patients with BCVI *and* CoW anomalies are needed as current guidelines do not provide recommendations for these treatments within this population.

While there was no difference in the stroke rate when compared by the anatomical region of the CoW affected in this study, those with posterior anomaly experienced more CSBs after antithrombotic initiation than those with anterior anomalies, which may indicate that those with posterior anomalies need varying antithrombotic regimens than those with anterior anomalies, such as lower antithrombotic doses, less frequent antithrombotic regimens, or increased monitoring of coagulation factors such as INR to direct treatment. In the study by Zhou et al., they found that those with posterior anomalies, or with both posterior and anterior anomalies, had significantly higher NIHSS on discharge than those with a normal CoW [48]. Shahan et al. found fetal-type enlarged persistent posterior communicating artery anomalies were associated with a trend towards a decreased stroke risk [17]. Only three patients in this study had posterior communicating artery anomalies, none of whom experienced a stroke.

Because of the association between CoW and poor outcomes, knowledge of factors associated with CoW anomalies may be useful for their identification. In this study patients with CoW anomalies had a significantly higher rate of having a prior MI and a lower rate of having pre-existing diabetes than patients with a normal CoW. In contrast to our study, another study of patients with cerebral infarctions observed that CoW anomalies were associated with an increased rate of having pre-existing diabetes when compared to patients with a normal CoW [48]. Chi et al. also studied a population of stroke patients and observed that diabetes was associated with strokes occurring specifically in the posterior vertebral basilar artery and posterior cerebral artery, rather than an infarction affecting vessels in the anterior circulation [51]. In this population of patients with BCVI, CoW anomalies occurred more frequently in the anterior arteries (*n* = 78) than the posterior arteries (*n* = 12), which may explain why these results differ from previous studies finding an higher rate of diabetes among patients with a CoW anomaly but had more anomalies in posterior vessels [48, 51]. This is the first study to our knowledge that has found an increased rate of prior MI among patients with BCVIs and CoW anomalies. In this study while diabetes and MI were significantly associated with CoW anomalies, the overall rate of diabetes (8%) and MI (< 1%) were low, and neither factor were identified as confounding variables as neither diabetes [0% - stroke in diabetics vs. 6% stroke in non-diabetics, *p* = 0.15] nor MI (0% -stroke among patients with prior MI vs. 5% - stroke in patients without prior MI, *p* > 0.99) were associated with the development of stroke in this population (data not tabulated).

Universal screening has been suggested because lengthy BCVI screening criteria are thought to be complicated and studies have found that many BCVIs (20–40%) do not meet screening criteria [9, 13, 16, 24]. Conversely, Müther et al. found 27% of BCVIs were still missed when screening all major trauma patients receiving CTAs [3]. Cook et al. reported that to identify all BCVIs, 96% of trauma patients would require screening [52]. Depending on how universal screening is implemented, some BCVIs still may be missed. Universal screening was not conducted at any of the participating centers but 38% of the index scans identifying a BCVI were conducted for trauma admission scanning, or because of some other head/neck injury not outlined as BCVI screening criteria. Furthermore, patients with CoW anomalies had scans due to some other head/neck injury not outlined as BCVI screening criteria significantly more than patients with a normal CoW. This provides evidence that expanding BCVI screening criteria to all head/neck injuries may improve diagnosis rates for both BCVI and CoW. Three BCVI scanning criteria were more common among patients with CoW anomalies (severe TBI with a GCS < 6, a complex skull fracture, or a mandible fracture); presence of these specific injuries associated with BCVI may be useful in prompting detailed imaging including the CoW to look for anomalies. However, these three criteria were notably not identified as significantly associated with the risk of stroke in this population (data not tabulated).

The CT-scanner configuration varies across guidelines; EAST recommends at least an 8-slice, Scandinavian Neurotrauma and WTA recommend at least a 16-slice, and the ACS recommends at least a 64-slice [14, 26, 27, 53]. In a meta-analysis, the sensitivity of CTAs compared with DSA did not improve when the scanner configuration was increased above 16-slice [29]. The most common scanner configuration used for the index BCVI scan and scans identifying a CoW anomaly was 64-slice. The 64-slice was available at all participating centers, and 64-slice was the highest option at seven of the 17 centers (41%); 128-slice was the highest at six centers (35%), (Supplemental Fig. 1). It appears that some centers defaulted to the highest scanner configuration available. Additionally, over time use of 16-slice significantly decreased, and use of 128-slice significantly increased (Supplemental Fig. 2). Thus, as scanners advance and higher scanner configurations are available, there may continue to be a trend in use of higher scanner configuration. While DSA is the gold standard, it is associated with higher costs and more complications than CTA, and CTA has shown comparable results in the detection of BCVI, these factors may explain why CTA was almost exclusively used in this study [3, 12, 14, 20, 26, 27, 29–31, 33, 34, 39]. The use of other screening modalities (MRI, MRA, and ultrasound) were very rare, and may be because prior studies found they have a lower accuracy, take longer to complete as they often require sedation, and have low sensitivity for low-grade BCVIs [6, 31, 35, 40]. 

Supplemental Fig. 2 describes trends in the scanner configuration use over time, summarized as proportions by the year of admission and the scanner configuration used. Symbols were used to differentiate the scanner configuration and mark the percentage of patients who had a scan during hospitalization with that corresponding scanner configuration. Linear trend lines were fitted for each scanner configuration. Asterisks next to the scanner configuration in the legend indicate a significant trend was observed over time for use of that scanner configuration. Over time there was a significant decrease in utilization of 16-slice CT (*p* = 0.04), and a significant increase in the utilization of 128-slice CT (*p* = 0.02). The use of other CT-slices [32-slice CT (*p* = 0.25), 64-slice CT (*p* = 0.80), and 512-slice CT (*p* = 0.86)] remained constant over time.

The region to scan for BCVI identification is not specified in the WTA or EAST guideline [27, 54]. Whereas, the ACS and the Scandinavian NeuroTrauma Guideline recommends to include the CoW by including the neck during BCVI scans [14, 26]. Hundersmarck et al. observed an increased BCVI incidence when adding the neck to their whole-body scan [2]. In this study, scans identifying a CoW anomaly were part of a whole-body scan less often than scans which did not identify a CoW anomaly. Whole-body scans are associated with false positives and undetermined grades, potentially because the patient’s arms are raised compromising the image quality [36, 55]. It is possible that scans as part of a whole-body scan may not adequately visualize the CoW as well. Scans of the head and neck only may be ideal for identification of CoW anomalies as patients with a CoW anomaly had scans of both the head and neck only significantly more often than patients with a normal CoW.

Aljuboori et al. state follow-up imaging is debated especially for high grade BCVIs which are unlikely to resolve [42]. Wu et al. observed that higher grade BCVI were more likely to have follow-up imaging but were less likely to improve than the lower grade injuries [38]. Alternatively, repeat scanning may be useful to direct treatment: antithrombotic therapy may be discontinued for resolving injuries and endovascular surgery can be considered for worsening grades [6, 42]. Future research on how repeat scans change treatment may be useful. In this study, a majority of patients with a repeat scan had no change in their grade and only 17% of patients grade worsened. Other studies have reported low rates of worsening BCVI grades (8–34%) [9, 21, 22, 42]. Progression varied by the artery involved, patients with carotid artery injuries showed changes in their grade, whereas vertebral injuries were more likely to have no change or to have an unknown progression. This may be because those with carotid artery injuries had grade I injuries (50–51%) on their index scan more often than vertebral artery injuries (36–37%). Cothren et al. also observed a high rate (78%) of grade I injuries among carotid artery BCVIs [46]. 

One notable finding on repeat scans was that 11% of patients had newly detected BCVIs on subsequent scans. While the BCVI grade directs treatment methods, and follow-up scans are thought to assist with treatment decisions, current guidelines do not make recommendations based on the number of arteries involved, or newly detected BCVIs appearing on subsequent scans [14, 26, 27, 53]. Future studies evaluating treatment efficacy for patients with newly detected BCVIs on subsequent scans are needed.

### Limitations

This was a retrospective study with no long-term follow-up. The scans that did not identify BCVI were not collected, thus scans that missed BCVI diagnoses and completely resolved BCVIs were not summarized. Screening criteria, scanning practices, and reporting of CoW anomalies were not standardized across the seventeen participating centers with varying ACS trauma designations. In general, the higher-level trauma centers were utilizing the expanded Denver criteria, while the lower-level trauma centers had differing screening criteria or scans may have been ordered at the treating physician’s discretion. The total number of patients screened for BCVI is not known, but the frequency of BCVI among all trauma patients admitted to the Level I participating centers was 1.1% (521/47,763) and was 0.2% (40/16,969) at the Level II-IV participating trauma centers. There was variation in the scanner configuration resolutions available at each center (Supplemental Fig. 1), physicians may have used the only scanner configuration or a standardized scanner configuration rather than deciding on the scanner configuration. Contrast practices (e.g., injection rate, timing), specific descriptions of CoW anomalies (e.g. missing, aneurysms, fetal-type variations, etc.) and specific details on CSBs were not collected. There was no formal power analysis conducted for the aims examining outcomes compared by presence of CoW anomalies, which was an aim of this larger descriptive study on scanning practices for BCVI and CoW anomalies. Because of the small number of patients with a CoW anomaly, adjusted analyses to account for confounding variables were not conducted. This study was conducted within a network of American College of Surgeons accredited trauma centers located in the United States; the results may not be generalizable to other hospitals which are not accredited trauma centers, or to hospitals situated in other regions. Further the results may not be generalizable to pediatric patients, or to other centers using less advanced or differing radiological diagnosis modalities than described in this study.

## Conclusions

Patients with CoW anomalies were more likely to suffer strokes, ICHs, and CSBs before antithrombotic initiation than those with a normal CoW. Those with posterior CoW anomalies were more likely to have CSBs after antithrombotic initiation than patients with anterior CoW anomalies. This highlights the need to include the CoW in BCVI imaging studies. Despite the low frequency of MI, it was significantly associated with presence of CoW anomalies. Severe TBIs with a GCS < 6, complex skull fractures, and mandible fractures were BCVI screening criteria that more common among patients with a CoW anomaly. The presence of these specific BCVI screening criteria, or BCVI with a prior MI, may be useful in prompting imaging to look for CoW anomalies. Roughly a third of the index scans identifying BCVI were conducted as part of the routine trauma admission scanning, or because the patient had some other head/neck indication not included in BCVI screening criteria. Patients with CoW anomalies were scanned because of some other head/neck indication not included in BCVI screening criteria significantly more often than patients with a normal CoW. Screening for BCVI across all patients with a head/neck injury may improve BCVI and CoW detection. Including the head and neck in BCVI imaging studies may assist in simultaneous diagnosis of strokes and CoW anomalies, which may not be visible on neck only scans. While changes to the BCVI grade were not common, newly detected BCVIs were diagnosed on follow-up imaging, suggesting the injuries may be worsening. This study had a relatively small sample size, limiting the ability to control for potential confounding variables. Future larger studies are needed and should focus on evaluating how and when follow-up imaging impacts treatment, and if patients with CoW anomalies or newly detected BCVIs would benefit from tailored treatment.

### Electronic supplementary material

Below is the link to the electronic supplementary material.

Supplementary Material 1

## Data Availability

The dataset(s) supporting the conclusions of this article are not publicly available per data use agreements with the participating centers. A limited dataset can be made available upon reasonable request.

## Abbreviations

BCVI: blunt cerebrovascular injuries
ACS: American College of Surgeons
TQIP: Trauma Quality Improvement Program
DSA: Digital subtracted angiography
CTA: computed tomography angiography
MRI: Magnetic resonance imaging
MRA: Magnetic resonance angiography
EAST: Eastern Association for the Surgery of Trauma
ICH: Intracerebral hemorrhage
FVCA: Four vessel cerebral angiography
HLOS: Hospital length of stay
ICULOS: Intensive care unit length of stay
COW: Circle of Willis
EHR: Electronic health records
SAS: Statistical Analysis System
SD: Standard deviation
IQR: Interquartile range
MVC: Motor vehicle collision
WTA: Western Trauma Association
CT: computed tomography
AHA: American Heart Association
CSB: clinically significant bleeds
