# Supplementary material for: The effect of circle of willis anatomy and scanning practices on outcomes for blunt cerebrovascular injuries
Source: Scand J Trauma Resusc Emerg Med. 2024 Jun 17;32:57. doi: 10.1186/s13049-024-01225-x (PMC11181559; doi:10.1186/s13049-024-01225-x)
Supplement: Supplementary file 1 — Supplementary Material 1 [file 13049_2024_1225_MOESM1_ESM.docx]

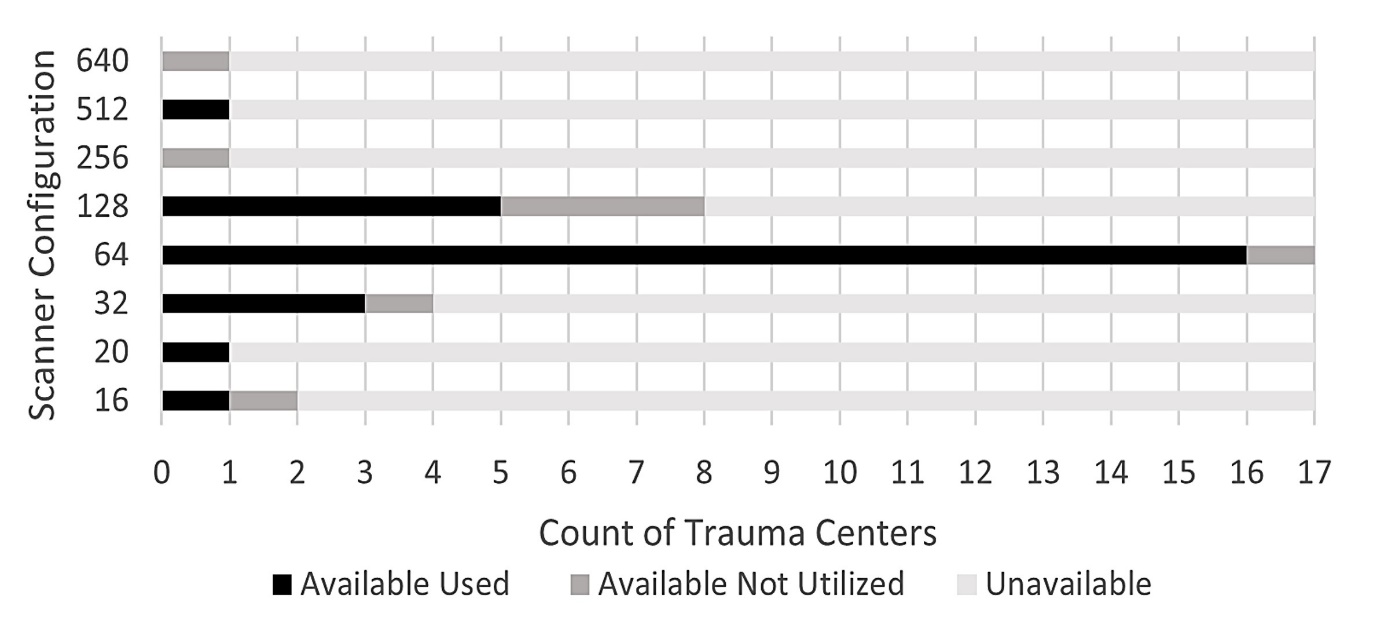

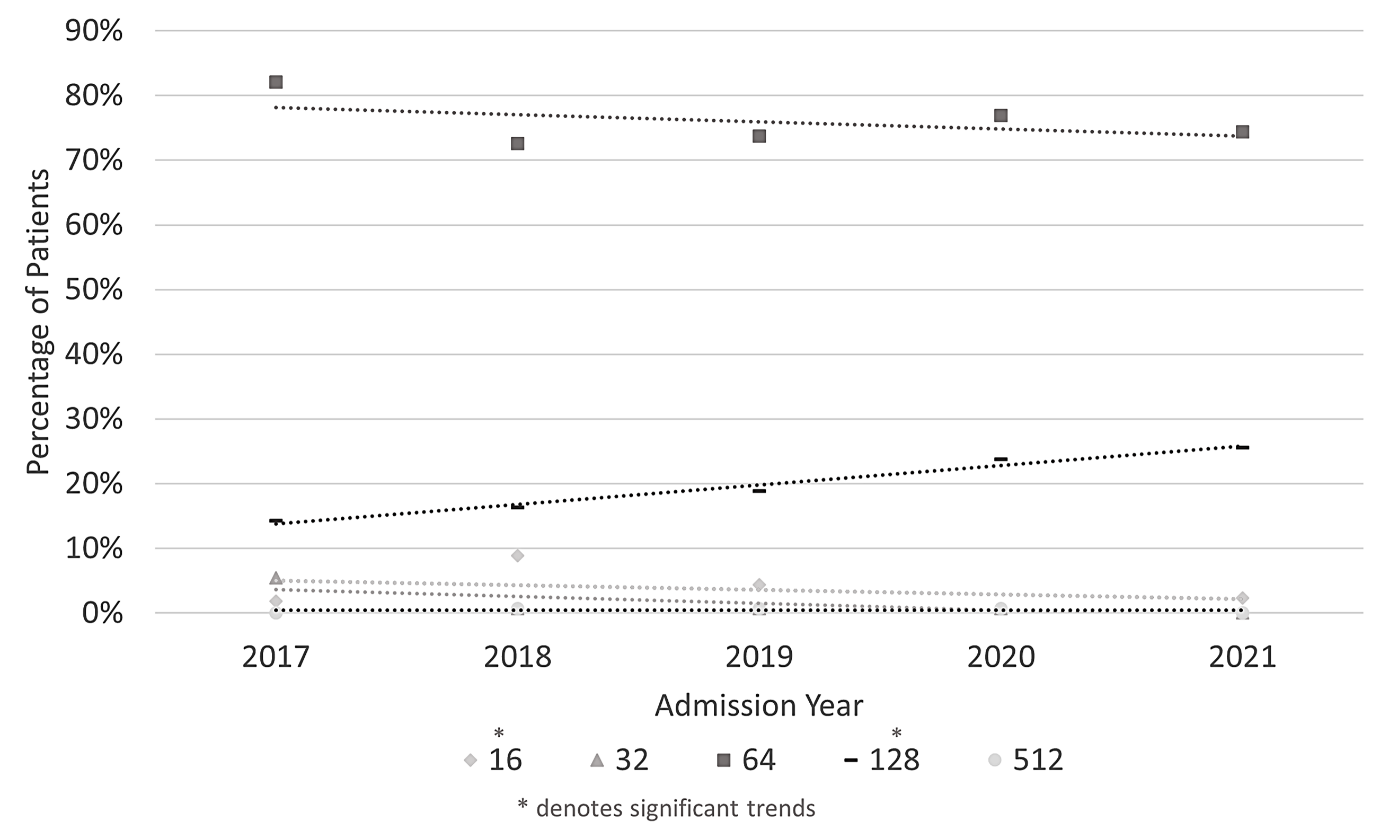


**Supplemental Figure 1**: Scanner Configuration and Utilization at the 17 Participating Trauma Center

**Supplemental Figure 2**: Trends in Scanner Configurations Over Time

| **Supplemental Table 1: BCVI Artery Involved and Grade** | | | | | |  |  |
| --- | --- | --- | --- | --- | --- | --- | --- |
|  | **Highest Grade Diagnosed** | **1st BCVI Diagnosis 100% (561)** | **2nd BCVI Diagnosis  37.1% (208)** | **3rd BCVI Diagnosis 7.8% (44)** | **4th BCVI Diagnosis  3.2% (18)** | | |
| **Left Carotid**, % of total BCVIs (n) | 25.3% (142) | 24.2% (136) | 26.4% (55) | 34.1% (15) | 33.3% (6) | | |
| Grade I, % of Left Carotid BCVIs (n) | 52.3% (68) | 51.4% (70) | 34.5% (19) | 13.3% (2) | 16.7% (1) | | |
| Grade II, % of Left Carotid BCVIs (n) | 13.8% (18) | 16.1% (22) | 9.1% (5) | 0% (0) | 0% (0) | | |
| Grade III, % of Left Carotid BCVIs (n) | 14.6% (19) | 12.5% (17) | 23.6% (13) | 33.3% (5) | 0% (0) | | |
| Grade IV, % of Left Carotid BCVIs (n) | 9.2% (12) | 6.6% (9) | 12.7% (7) | 26.7% (4) | 33.3% (2) | | |
| Grade V, % of Left Carotid BCVIs (n) | 2.3% (3) | 1.5% (2) | 0% (0) | 13.3% (2) | 0% (0) | | |
| Grade UTD, % of Left Carotid BCVIs (n) | 7.7% (10) | 11.0% (15) | 20.0% (11) | 13.3% (2) | 50.0% (3) | | |
| **Right Carotid**, % of total BCVIs (n) | 26.2% (147) | 24.8% (139) | 27.9% (58) | 29.5% (13) | 33.3% (6) | | |
| Grade I, % of Right Carotid BCVIs (n) | 51.1% (67) | 50.4% (70) | 37.9% (22) | 15.4% (2) | 16.7% (1) | | |
| Grade II, % of Right Carotid BCVIs (n) | 19.8% (26) | 20.9% (28) | 15.5% (9) | 23.1% (3) | 0% (0) | | |
| Grade III, % of Right Carotid BCVIs (n) | 16.0% (21) | 12.2% (17) | 15.5% (9) | 15.4% (2) | 33.3% (2) | | |
| Grade IV, % of Right Carotid BCVIs (n) | 3.8% (5) | 3.5% (5) | 5.2% (3) | 23.1% (3) | 16.7% (1) | | |
| Grade V, % of Right Carotid BCVIs (n) | 1.5% (2) | 1.4% (2) | 0% (0) | 0% (0) | 0% (0) | | |
| Grade UTD, % of Right Carotid BCVIs (n) | 7.6% (10) | 12.2% (17) | 25.9% (15) | 23.1% (3) | 33.3% (2) | | |
| **Left Vertebral**, % of total BCVIs (n) | 44.4% (249) | 42.8% (240) | 43.8% (91) | 36.4% (16) | 44.4% (8) | | |
| Grade I, % of Left Vertebral BCVIs (n) | 36.6% (85) | 36.3% (87) | 27.5% (25) | 31.3% (5) | 25.0% (2) | | |
| Grade II, % of Left Vertebral BCVIs (n) | 27.2% (63) | 28.3% (68) | 17.6% (16) | 0% (0) | 0% (0) | | |
| Grade III, % of Left Vertebral BCVIs (n) | 5.2% (12) | 4.2% (10) | 8.8% (8) | 6.3% (1) | 12.5% (1) | | |
| Grade IV, % of Left Vertebral BCVIs (n) | 21.1% (49) | 20.4% (49) | 27.5% (25) | 31.3% (5) | 25.0% (2) | | |
| Grade V, % of Left Vertebral BCVIs (n) | 2.2% (5) | 2.1% (5) | 1.1% (1) | 0% (0) | 0% (0) | | |
| Grade UTD, % of Left Vertebral BCVIs (n) | 7.8% (18) | 8.3% (21) | 17.6% (16) | 31.3% (5) | 37.5% (3) | | |
| **Right Vertebral**, % of total BCVIs (n) | 40.5% (227) | 39.2% (220) | 43.3% (90) | 43.2% (19) | 44.4% (8) | | |
| Grade I, % of Right Vertebral BCVIs (n) | 37.2% (77) | 37.3% (82) | 28.9% (26) | 15.8% (3) | 12.5% (1) | | |
| Grade II, % of Right Vertebral BCVIs (n) | 28.5% (59) | 27.3% (60) | 28.9% (26) | 5.2% (1) | 0% (0) | | |
| Grade III, % of Right Vertebral BCVIs (n) | 3.9% (8) | 2.7% (6) | 4.4% (4) | 15.8% (3) | 25.0% (2) | | |
| Grade IV, % of Right Vertebral BCVIs (n) | 19.8% (41) | 19.5% (43) | 23.3% (21) | 31.6% (6) | 25.0% (2) | | |
| Grade V, % of Right Vertebral BCVIs (n) | 1.4% (3) | 1.4% (3) | 0% (0) | 0% (0) | 0% (0) | | |
| Grade UTD, % of Right Vertebral BCVIs (n) | 9.2% (19) | 11.8% (26) | 14.4% (13) | 31.6% (6) | 37.5% (3) | | |
| **Multiple Arteries Involved,** % (n) | 26.9% (151)* | 23.5% (132) | 29.8% (62) | 25.0% (11) | 27.8% (5) | | |
| BCVI: blunt cerebrovascular injury, UTD: unable to determine. *This proportion (n) displays the patients with multiple artery abnormalities across all patients, on any scan. Two patients received a fifth BCVI scan, with a total of 4 BCVIs diagnosed (left carotid unknown (n=1), right carotid unknown (n=1), right vertebral grade IV (n=1), and right vertebral unknown (n=1). Carotid artery injuries could have occurred anywhere along the cervical carotid artery: the common, external, or internal carotid artery. | | | | | | |  |
